# Social Reintegration Experiences of Young Adult Cancer Survivors

**DOI:** 10.3390/bs14111101

**Published:** 2024-11-15

**Authors:** Ji Seong Yi, Song Yi Lee

**Affiliations:** Department of Counselling and Coaching, Dongguk University-Seoul, 30, Pildong-ro 1 gil, Jung-gu, Seoul 04620, Republic of Korea; 2020126709@dgu.ac.kr

**Keywords:** cancer, survivors, young adult, social reintegration, perception, Q methodology

## Abstract

This study uses the Q methodology to investigate the subjective perceptions of social reintegration among cancer survivors in their 20s and 30s. We organised a Q population through a pilot study and interviews and finalised 40 Q sample items. For P sample representativeness, we used purposive sampling and selected 12 individuals by age and cancer type. After a Q sorting process, we conducted a key factor analysis using Ken-Q Analysis Desktop Edition. We identified four types of P samples based on their perceptions and noted the main characteristics of each type. We characterised Type 1 as “recovery of presence through social reintegration seeking”, Type 2 as “confusion in social reintegration due to social prejudices”, Type 3 as “psychosocial support in the process of social reintegration”, and Type 4 as “blessing in disguise for post-traumatic growth”. The results suggest a need for practical and institutional support reflecting cancer survivors’ characteristics by type. This study provides basic data that researchers could use to develop coaching and counselling services to support the social reintegration of cancer survivors in their 20s and 30s.

## 1. Introduction

Cancer is the second leading cause of death globally [1], and in 2023, the National Statistical Office [2] announced that it was the primary cause of death in South Korea. Data from the National Health Insurance Service [3] indicates that newly registered cancer patients increased by 43% in 2021 compared to 2014. However, the survival rate is also increasing. According to 2021 National Cancer Registration Statistics [4], the five-year relative survival rate (“survival rate”) of cancer patients from 2017 to 2021 was 72.1%, with at least 70% of patients surviving for at least five years [4].

Based on such trends, in South Korea, terms such as “cancer experiencer” and “cancer survivor” have become common beyond cancer patients, and various discussions are still in progress regarding the terminology [5]. The National Coalition for Cancer Survivorship, established in 1986, defined “cancer survival” as the experience of living after a cancer diagnosis [6] and “cancer survivors” as all persons who remain alive after a cancer diagnosis. Meanwhile, Article 12, paragraph 2, of the Cancer Control Act of South Korea defines a “cancer survivor” as a non-terminal cancer patient under Article 2, paragraph 3 of the Act on Hospice and Palliative Care and Decisions on Life-sustaining Treatment for Patients at the End of Life Process [7].

Despite increased cancer survival rates, people still treat cancer as a sudden, life-threatening traumatic event [8]. In addition, while cancer was previously limited to diagnosis and treatment, it is now a chronic disease that requires continuous management, and there is much interest in the quality of life of cancer survivors [9,10]. In 1995, the South Korean government established the first ten-year plan to mitigate the negative effects of cancer by constructing a national cancer management system [11]. Then, in 2016, through the Third Comprehensive Cancer Control Plan, the government presented an expanded policy goal to construct an integrated support system for cancer survivors [12]. In addition, in 2021, the government presented the Fourth Comprehensive Cancer Control Plan to support cancer survivors as they return to society. This initiative was crucial in constructing equal cancer management bases [13].

Since 1995, the incidence rate of cancer patients in their 20s and 30s has increased rapidly and steadily. Statistics [14] show that 25,384 individuals in their 20s and 83,944 in their 30s were diagnosed with cancer in 2021. In addition, from 2016 to 2021, the incidence rate of cancer among those in their 20s increased substantially to 26%, indicating that individuals in their 20s and 30s are no longer safe from cancer [14].

Moreover, according to 2022 data from the National Statistical Office [15], life expectancy, defined as the average number of years a person will live, was 82.7 years. The Ministry of Health and Welfare announced that the average age with high cancer incidence rates is 60–64 years [16]. If the average life expectancy is roughly 83 years, someone diagnosed with cancer at the age of 60 could live with cancer for approximately 20 years, and cancer patients in their 20s or 30s may live as cancer survivors for over 50 years.

People who become cancer survivors in their 20s and 30s begin to experience a variety of cancer treatment-related psychosocial sequelae. This situation disrupts their physical, cognitive, emotional, and social development [17]. Research shows that the risk of the occurrence of secondary primary malignancy after treatment is high [18], and the possibility of infertility increases in young cancer survivors [19]. Another study showed that compared to that in a control group with no history of cancer, post-traumatic stress was high in male and female cancer patients, with depression and anxiety being particularly high in women [20]. In addition, practical barriers to returning to school and work emerged due to cognitive and emotional problems [21,22]. Research also shows that cancer survivors experience uncertainty about employment, and their treatment may disrupt their social relationships, leading them to feel alienated [23,24].

On the other hand, many studies have reported positive psychological, cognitive, and behavioural changes in cancer patients despite negative outcomes [25,26]. Scholars refer to such changes as “post-traumatic growth”, which is a common phenomenon in young cancer survivors. It appears most prominently in relationships with family members and peers and in survivors’ plans and goals. Over time, post-traumatic growth reduces the pain caused by trauma [27,28]. Greup et al. [29] stated that post-traumatic growth positively affects life satisfaction. However, post-traumatic growth does not necessarily mean adaptation [30], and harmonising one’s new life circumstances—adapting well through social reintegration—is important [26,30,31,32]. Returning to daily life after treatment and achieving social reintegration can be very challenging for young cancer survivors [17].

Understanding the social reintegration of cancer survivors in their 20s and 30s requires an exploration of the developmental tasks and characteristics that define young adulthood. Havighurst [33] stated that humans must accomplish certain tasks—which he named “developmental tasks”—at different stages of their lives. Levinson [34] classified the period from 22 to 40 years as early adulthood and explained that this period encompasses much exploration and many choices. This stage is when people, for example, set life goals, choose jobs, and become members of society. Erikson [35] said that in the early stages of adulthood (the 20s and 30s), one needs to form close relationships with others based on deep empathy and understanding, and that people feel isolated when they cannot form such relationships [36,37].

This period highlights a societal expectation for people to define their identities, establish personal and career goals, achieve financial independence through employment, and often marry and establish their households [38]. Arnett [39] viewed this period as an important part of a person’s development because it is when a person sets up their future. However, cancer survivors in their 20s and 30s face unique challenges as the disease disrupts their accomplishment of key developmental tasks. After completing active treatment in the hospital, they must navigate the difficult process of resuming these tasks while reintegrating socially. They must face a new life requiring continuous readjustments [40].

“Social rehabilitation” derives from the term “rehabilitation” and refers to the process by which persons with acquired disabilities return to normal social life by maximally recovering their physical, mental, social, occupational, and economic abilities through education and training [41]. The U.S. National Institute of Health [42] defines “social rehabilitation” as the recovery of abilities lost due to disease or treatment to function normally or nearly normally. Ore et al. [43] used “reintegration” to refer to a cancer survivor’s return to society. This process involves helping people return to their previous living environments after enduring an illness or disease and includes overcoming difficult environments and social situations [44]. In medicine and related health fields, a person’s reintegration into normal living means harmonising their physical, psychological, and social characteristics [45].

Thus, this study conceptualises reintegration as a return to society. The cancer experience can be a resource for returning to society rather than shifting from abnormality to normality. Reintegration means gathering the elements that make up the past and present to recreate a whole, intact person. It also means that an individual can do as they please. Moreover, the concept extends beyond functional status to include physical and psychosocial elements [43]. Resuming socio-economic activities through social reintegration is crucial to helping cancer survivors improve their self-esteem, restore normalcy as members of society, and attain a stable quality of life through economic security [9,46,47]. Chae and Song [48] stated that cancer survivors strongly desire social reintegration after treatment, especially regarding economic activities, such as returning to work or finding new jobs.

Through the Cancer Control Act, the state is establishing and implementing comprehensive policies for cancer prevention, treatment, and research. Specifically, it is operating central and regional integrated support centres for cancer survivors under Article 12-2 (Cancer Survivors Integrated Support Project) of the Cancer Control Act to promote projects that improve the health of cancer survivors and their families. These efforts also intend to promote school and social reintegration. However, the Cancer Survivor Integrated Support Project and the Cancer Survivor Integrated Support Center present an unclear definition of “social reintegration”, as they use this term interchangeably with “return to work” [49,50]. In addition, the socio-economic area supported by the Cancer Survivor Integrated Support Center lacks concrete services related to vocational rehabilitation [48]. Therefore, patients require various services for social reintegration [9,51,52].

Previous studies on the social reintegration of cancer survivors in South Korea lack diversity, as they concentrate on certain cancer types, especially breast cancer [11]. Therefore, scholars have reported the need for studies on various types of cancer and cancer survivors in different age groups [53]. Lee Si Eun [54] revealed that workers’ perceptions of cancer, cancer survivors, and cancer survivors’ return to work had not changed substantially over ten years. In addition, studies have analysed cancer survivors’ experiences in returning to work [46,55,56,57,58], return-to-work intervention programmes [59], unmet needs of maintaining a job, and cancer survivors’ experiences of returning to daily life activities [60,61]. Most studies relate to cancer survivors’ return to work; the only study related to social reintegration was “A Study on Cancer Survivors’ Experience of Job Hunting and Social Reintegration Using Focus Group Interviews” conducted by Kim Miran [62].

Young adult cancer survivors face long-term physical and psychological challenges [63,64]. These effects can last for years after survival and affect many areas of life, including quality of life, intimate relationships, finances, and career. In addition, young cancer survivors are often at a higher risk of long-term effects than paediatric, middle-aged, and older cancer survivors [65]. Thus, to prevent and address the unique and complex psychosocial problems of young cancer survivors, it is necessary to provide individualised and specific methods for the social reintegration of young cancer survivors in their 20s and 30s [66]. This approach requires a study on the subjective perceptions of social reintegration of cancer survivors in their 20s and 30s; however, scholars have not conducted such a study to date.

In addition, the social reintegration of cancer survivors leads to a lifelong survival process, and survivors’ experiences can differ depending on their sociocultural contexts [43]. Cancer survivors in their 20s and 30s may have very different perceptions of social reintegration than older cancer survivors. Thus, we need studies on this group’s subjective perceptions to comprehend their specific experiences of social reintegration [67,68]. Therefore, this study used the Q methodology to measure this group’s subjective attitudes to understand their perspectives on their social reintegration experiences [69]. This study also provides useful basic data for developing coaching and counselling services that support the social reintegration of this group by categorising their perceptions of social reintegration and examining their characteristics.

Based on the above, the research questions (RQs) addressed in this study are as follows:

RQ1. What are the perceptions of social reintegration among cancer survivors in their 20s and 30s?

RQ2. What are the characteristics of different perceptions of social reintegration among cancer survivors in their 20s and 30s?

## 2. Materials and Methods

British physicist and psychologist Stephenson [70] created the Q methodology to measure and systematically study various aspects of the subjectivity of humans, such as their attitudes, beliefs, and values. The Q methodology provides a basis for systematic studies by uncovering people’s subjective structures, attitudes, and perspectives [71]. Stephenson [70] described the strength of the Q methodology as “the science of subjectivity” because it combines quantitative and qualitative data and analysis techniques [72]. Although the Q methodology follows existing factor analysis, it is unique in that “value objects” (item statements) are the independent variable, and “subjects” (individuals, respondents, survey subjects) are the dependent variable. In this way, the Q methodology groups humans into factors and extracts these factors into types [73].

There are limitations to studying individuals’ subjectivity through existing quantitative methods, and scholars might overlook aspects following quantitative procedures [74,75]. However, the Q methodology allows individuals to express their subjectivity towards objects, focusing on capturing participants’ subjective experiences and understanding [71,76]. Thus, researchers can understand subjects’ perspectives and reveal marginalised or unknown and potentially hidden perspectives [77,78]. In this respect, the Q methodology fits the purpose of the present study, which explores and categorises the subjective perceptions of social reintegration among cancer survivors in their 20s and 30s.

This study followed the procedures illustrated in Table 1.

### 2.1. Q Population Organisation

The Q methodology’s foundation is the “concourse”, a technical concept that refers to the field in which humans communicate their subjectivity [79]. Researchers often use a concourse in the Q methodology to collect all possible statements from a respondent about the relevant topic. Therefore, a concourse must include all aspects of the relevant discourse. A concourse can consist of self-referent statements (i.e., opinions rather than facts), objects, photos, or other elements. Researchers can collect oral statements through various methods, including interviews, participant observations, media reports, popular literature such as newspapers, magazines, and novels, and scientific literature such as papers, essays, and books. The collected data become the study’s raw material [80].

This study selected the Q population as follows. We collected the Q population by examining seven literature surveys such as theses, journals, and research reports related to the social rehabilitation of young cancer survivors in the 2030s, including ‘2030s cancer patients’, ‘cancer survivors’, and ‘social rehabilitation of cancer survivors’, and the following titles: “Cancer Survivors’ Life Experience” [81], “The Influencing Factors on Quality of Life among Breast Cancer Survivors” [82], “Integrative Review of Guidelines Related Symptom Management and Physical Activity for Developing of Self-Care Management Programme for Cancer Survivors” [83], “Recognition of Nursing College Students About General Cancer and Cancer Survivors Returning to Work” [84], “Cancer Survivor’s Job Search and Social Return Experience Using a Focus Group Interview” [62], “The Experience of Cancer Survivor’s Return to Everyday Life” [61], and “Qualitative Research on the Experiences after Cancer Diagnosis of Unmarried Female Breast Cancer” [85].

We also obtained 63 statements through internet search engines such as Google, Naver, and Daum. In the second phase of data collection, we conducted in-depth interviews with semi-structured questions. In-depth interviews are an important process in the Q-methodology because they can elicit the views of research participants in-depth [86]. We selected interviewees considering theoretical rather than statistical representation in quantitative research, and scholars have stated that four interviewees are sufficient for the Q method in relation to the research topic [86,87]. Therefore, in this study, we selected cancer survivors in their 20s and 30s, considering the stage and type of cancer in relation to the research topic. Thus, after explaining the purpose of the study and obtaining their consent, we interviewed four individuals: one woman in her 20s (breast cancer, stage 1), two women in their 30s (lobular sarcoma cancer, stage 2; salivary gland cancer, stage 3), and one man in his 30s (thyroid cancer, stage 2).

The interview questions comprised the following:

- At what age were you diagnosed with cancer?
- What type of cancer was it?
- What stage was the cancer?
- How long did the treatment take?
- How many years have passed since you received hospital treatment?
- If you were to compare your social reintegration experience before and after being diagnosed with cancer, what would be the biggest difference?
- What words come to mind when you think of social reintegration?
- What was your social reintegration experience?
- Do you have any memorable experiences of the social reintegration process?
- What was the most difficult part of the social reintegration process?
- What was helpful in the social reintegration process?
- What is the most important factor for reintegrating cancer survivors in their 20s and 30s into society?
- What else would you like to add?

Each interview lasted approximately 50 min, and we conducted in-depth interviews until saturation. Through this process, we added 45 statements to form a Q population of 108 statements.

### 2.2. Q Sample Selection

Q samples are statements to which participants apply Q sorting (i.e., the study subjects sort the statements) [75,88]. We initially categorised statements with common meanings or values to select Q samples. These statements underwent multiple re-categorisation processes to account for differences and ensure representativeness. Ultimately, researchers should choose the statements representing each category as the final Q samples [89].

This study implemented an unstructured sampling method appropriate for the research topic, with the validity of the Q sample verified three times. We collected 108 statements and documented and organised them in an Excel file. Then, based on the definitions and characteristics of social rehabilitation identified in previous studies, we categorised the statements from the Q population into the following areas: economic/occupational, social/relationship, physical, and psychological. Subsequently, we divided them into positive, negative, and neutral categories. We repeatedly reviewed and refined the organised statements through editing and removed duplicates and ambiguous questions. Through this process, we finalised the Q sample, resulting in a selection of 40 questions.

We then checked the statements’ appropriateness and understandability with one cancer survivor in his 20s and one in his 30s. We also conducted a pretest with three Q methodology experts. Pretests are necessary to verify participants’ understanding of and responses to the Q samples and instructions in advance so that Q sorting can proceed efficiently [75]. This process informed the Q sample sorting procedure, enabling participants to sort the Q samples. After the sorting process, we conducted interviews to determine whether the participants had difficulties with the classification process, whether the explanation of the guidance process was appropriate, and whether the statements were easy to understand. We modified and supplemented some items based on the interviews, ultimately selecting 40 Q statements.

### 2.3. P Sample Organisation

P samples are the subjects that sort the Q samples (i.e., the items) [69]. Researchers select many individuals as samples when following the R methodology, but the Q methodology requires a small sample size. This difference is because the Q methodology deals with intraindividual differences in significance rather than interindividual differences [69]. That is, the Q methodology does not infer the characteristics of all individuals using the proportion of people belonging to a certain factor. Instead, it identifies the structure to determine an individual’s interpretation of an object’s meaning [89].

The P samples do not require demographic representativeness because the Q methodology mainly aims at demographic generalisation [90]. In addition, according to the Small Sample Doctrine, the number of P samples should be small since the characteristics of types are not clear, and statistical problems may occur if there are too many P samples [91].

Researchers typically use 10 to 30 P samples [92]. This study used purposive sampling to select 12 young adult cancer survivors in their 20s and 30s as the P sample. We drew from sources such as online community bulletin boards for cancer patients and social enterprises for individuals with cancer experience in alignment with the study’s purpose. The selection criteria focused on cancer survivors in their 20s and 30s who are actively in the process of reintegrating into society, are undergoing curative treatment following a cancer diagnosis, or have completed major treatments (e.g., surgery, chemotherapy, radiation therapy). We ensured representativeness within the P sample by categorising participants by age and cancer type.

### 2.4. Q Sample Sorting

Q sorting involves selecting individuals as P samples and arranging the Q sample statements in order of importance based on their perspectives and conditions [75]. During Q sample sorting, the P samples read and sort individual statements using a forced normal distribution arrangement method [93].

The Q sorting stages utilised in this study are as follows. First, we asked the participants to become well-acquainted with the instructions, and then they read the 40 statement cards selected as Q samples. After that, we asked the participants to sort the statement cards based on their perceptions. Participants sorted the Q samples into three levels—positive, neutral, and negative—according to their degree of agreement.

**Figure 1:**
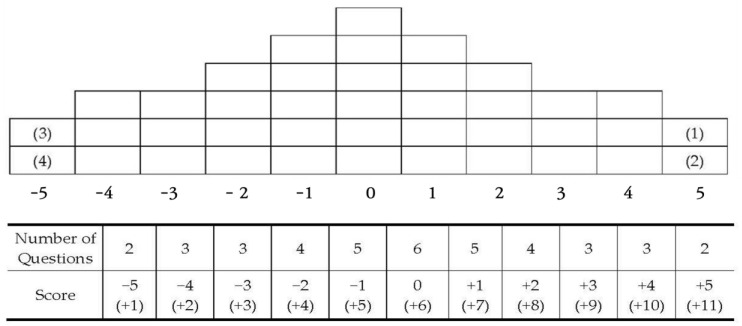
Q sorting distribution chart.

Next, we asked the participants to place the Q sample statements in the appropriate category on the sorting chart, ranging from −5 (for the statements with which they disagreed the most) to +5 (for those with which they agreed the most) (see Figure 1). The participants placed the statements with which they agreed the most on the rightmost position and those with which they disagreed the most on the leftmost position. They then organised neutral statements by filling in the positions from the outside to the inside of the sorting chart.

After the Q sorting process, we instructed the participants to review all the arranged cards. They verified that the arrangement matched the Q sort distribution. The participants could change their arrangement before attaching the cards to the sorting paper if needed. In addition, we asked the participants to provide their reasons for selecting the statements with which they most strongly agreed and disagreed.

After sorting the Q sample, we interviewed the participants to determine why they selected the statements. Such interviews enhance the richness of the data and improve the qualitative levels [94]. Q sample sorting took approximately 30–40 min per participant; we gave participants coffee coupons as a token of appreciation.

### 2.5. Data Analysis

We analysed data using KADE (Ken-Q Analysis Desktop Edition), a programme for Q sorting data analysis. We sequentially scored the data collected from the Q sorting from the most disagreeable statement (−5) to the most agreeable statement (+5) using the statement numbers shown in the Q sorting distribution chart. After coding in Microsoft Excel, we entered and analysed the data with KADE. This study conducted principal component factor analysis with Varimax rotation, a method that maximises the variance between factors to achieve the simplest structure [95]. To identify results that best explain each recognition type, we selected the final four types based on an eigenvalue of 1.0000 or higher. The eigenvalue serves as a criterion for determining types, with values of 1.0000 or greater set as the cutoff point [73], as scholars consider values below 1.0000 insufficient for reliably extracting and maintaining factors [96]. Upon completing factor rotation, we calculated the factor score (actor score) between each Q statement and factor. We identified Q statements with extreme values for each factor as those demonstrating the distinct characteristics of each factor and used the Z-score to select relevant items [86]. Additionally, we identified statements with which participants most agreed and most disagreed, focusing on subjects with high factor loadings in each type. We used the rationale for selecting these statements to interpret the characteristics of each type.

## 3. Results

### 3.1. Result Analysis

We derived four types (see Table 2). The eigenvalues were greater than 1 for all types (3.522128 for Type 1, 1.788463 for Type 2, 1.528208 for Type 3, and 1.040372 for Type 4). The cumulative explanatory power was 66%.

The correlations between the four types are shown in Table 3. A correlation coefficient indicates the degree of similarity between types, with a relevant coefficient close to 0 indicating an independent relationship [75]. The correlation coefficients between the types were as follows: 0.044 between Type 1 and Type 2, 0.1962 between Type 1 and Type 3, 0.3267 between Type 1 and Type 4, 0.2724 between Type 2 and Type 3, 0.1791 between Type 2 and Type 4, and 0.3686 between Type 3 and Type 4. Since the Q methodology does not assume complete independence between factors but instead focuses on discovering factors, no problems emerged regarding factor extraction methods depending on the value of the correlation coefficient [75].

The analysis of the P sample factors resulted in 12 participants, sorted into four types: four in Type 1, two in Type 2, three in Type 3, and three in Type 4. The demographic characteristics and factor weights of the P sample are shown in Table 4.

The standard score distribution and ranking for each type of Q statement are shown in Table 5. For the standard scores, positive values indicate agreement with an item, while negative values indicate disagreement. The greater the absolute value of a score, the stronger the level of agreement or disagreement. By examining the standard score of each Q statement for each type, we can explain the characteristics and personality traits of each type and confirm the subjectivity of research participants associated with that type.

### 3.2. Characteristics of the Types of Perceptions

#### 3.2.1. Type 1: Recovery of Presence Through Social Reintegration Seeking

Type 1 is the “recovery of presence through social reintegration seeking” type. Type 1 participants actively hoped for social reintegration, particularly in the context of returning to work. They believed there is an urgent need for institutional supplementation for social reintegration, and that social reintegration is a new challenge that entails restoring normality as a member of society. In addition, they hoped to regain their presence through social reintegration. On the other hand, ambivalence about social reintegration due to fear of cancer recurrence and metastasis was evident.

Per Table 5, Type 1 participants showed the strongest agreement with Q23 (“I am afraid that if I get stressed after social reintegration, the cancer will relapse or metastasise”) (z = 2.077), followed by Q22 (”Institutional supplementation to enable social reintegration is desperately necessary”) (z = 1.705), Q40 (”I would like to prove myself thinking that ‘I am still okay’ while working”) (z = 1.683), and Q37 (”I have an ambivalent feeling that I can do it, but I cannot do it (return to work, a new challenge, etc.))” (z = 1.362). Conversely, they showed the strongest disagreement with Q27 (“My confidence in my appearance is dropping”) (z = −1.607), followed by Q36 (”I feel really hopeless because there seems to be no hope”) (z = −1.59) and Q31 (“I feel like I was newly born again”) (z = −1.589).

P10 (8.90678), representing Type 1 with a high factor weight, agreed the most with statement Q22, explaining:

I am submitting my resume and going for interviews these days to pursue social reintegration. I have not written that I am fighting the disease in any of the documents I have been submitting these days, and I am emphasising that I am a very passionate person who wants to work. Last year, not long after the completion of my treatment, I disclosed that I had the disease because I had to visit the hospital frequently, and I could not get a job even though my document score was very high. I felt that just as companies should mandatorily hire people with disabilities, a system should also be established to require companies to hire people who have diseases but have the professional knowledge and skills needed to work.

P2 (6.64967) agreed the most with Q40, stating:

Given the times when I was lethargic during treatment, the confidence I gained while working was very good for recovering my self-efficacy. I am much happier and feel better now than when I was off work for treatment. I frequently take on challenges that seem a little reckless because I want to set a good precedent for those who suffer the same pain as me.

On the other hand, P3 (10) disagreed the most with Q31, stating:

If a person was born again, her life after fighting cancer should be changed to be more positive than her life before fighting cancer. But since my life before fighting cancer was more satisfying and better, I disagree the most with the relevant sentence. This is because the sadness, pain, and depression that I would not have had to experience if it had not been for the cancer diagnosis were great.

#### 3.2.2. Type 2: Confusion in Social Reintegration due to Social Prejudices

Type 2 is the “confusion in social reintegration due to social prejudices” type.

Type 2 participants received considerable help from their families, from their cancer diagnosis through the treatment process, and were very aware of the preciousness of family. On the other hand, in terms of social relationships beyond the family, they had concerns about negative perceptions and prejudices about cancer, which led them to worry about the social reintegration process and adaptation. In addition to being concerned and worrying about social prejudice, they were ambivalent about wanting to be actively engaged in society. However, rather than excluding themselves from society, they wanted to feel a sense of belonging.

Per Table 5, Type 2 participants showed the strongest agreement with Q11 (“I came to know the preciousness of family”) (z = 1.982), followed by Q19 (“I wish people did not know the fact that I was sick”) (z = 1.876), Q8 (“I am worried about whether I should reveal my cancer history or hide it when I resume my work”) (z = 1.692), and Q16 (“I want to date and get married, but it seems difficult”) (z = 1.586). Conversely, they showed the strongest disagreement with Q26 (“I am experiencing psychological difficulties due to infertility and decreased fertility”) (z = −1.876), followed by Q25 (“I have difficulties with my sex life, but there is no place where I can get help or talk about it comfortably”) (z = −1.876), and Q4 (“I envy my friends who are building their careers while working at companies”) (z = −1.48).

P6 (28.07677), representing Type 2 with a high factor weight, agreed the most with statement Q19. She explained, “I don’t want to tell people around me, especially those at the company I work for, that I was diagnosed with cancer because of the negative attention that comes with the word ‘cancer”. In addition, P4 (10.23293) agreed the most with statement Q8:

I am worried about whether or not I should reveal that I experienced cancer during an interview when joining a company. However, when I think about starting work again, I begin to think about issues such as using annual leave because I have to visit the hospital once a month and eating and drinking when I go to company dinners, etc. But I hesitate because I am unsure whether it would be a good idea to disclose my cancer. I also hate it because I think that after they find out about my cancer history, they will look at me with pity, or they will whisper among themselves about how to treat me.

P6 (28.07677) and P4 (10.23293) agreed most strongly with statement Q11. P6 said, “*I felt the importance of family at every moment,*” while P4 stated,

From the initial diagnosis through the treatment process, I often wondered how I would have managed on my own without my family. Just having the support of family is a tremendous help. You can share your feelings with friends, but sometimes you worry they might not want to keep hearing about your struggles if you continue to feel vulnerable. With family, however, it is comforting to be able to talk openly about your symptoms. But I also feel a sense of guilt.

On the other hand, P4 (10.23293) disagreed the most with Q17, stating:

After the surgery, I worked out diligently, pursued various hobbies, met new people, and overcame this challenging period with a lot of support and encouragement from my friends. Although I did not want to work in a company, I felt the need for a sense of belonging, so I took a part-time job packing flowers for four hours twice a week. As my boss recognised my efforts, I realised that I could be someone who was needed somewhere, and I started working harder with a sense of responsibility to become someone who could be of help. This experience made me believe that I could do it, too, so I did not feel left out of society.

#### 3.2.3. Type 3: Valuing Psychosocial Support in the Process of Social Reintegration

Type 3 is the “valuing psychosocial support in the process of social reintegration” type. Type 3 participants valued the process rather than the outcome of social reintegration. They believed that mental care is necessary for psychological stability, such as stress relief, emotional control, and growth, to manage uneasy feelings about social reintegration. In addition, this type highly valued psychological support experienced through relationships with family members and significant others.

Per Table 5, Type 3 participants showed the strongest agreement with Q38 (“I need mental care”) (z = 1.978), followed by Q23 (“I am afraid that if I get stressed after social reintegration, the cancer will relapse or metastasise”) (z = 1.764), Q31 (“I feel like I was newly born again”) (z = 1.691), Q11 (“I came to know the preciousness of family”) (z = 1.552), and Q12 (“I am encouraged by the consideration and support of those around me”) (z = 1.529). On the other hand, they showed the strongest disagreement with statement Q15 (“I feel lonely because there is no place to get help”) (z = −2.014), followed by Q1 (“I feel anxious because I am not able to do economic activities properly compared to my peers”) (z = −1.94) and Q2 (“I feel pathetic for having to rely on my parents at an age when I should be independent”) (z = −1.63).

P1 (10.35289), representing Type 3 with a high factor weight, agreed the most strongly with statement Q11. She gave the following reason for her selection:

The people on whom I relied the most during my fight against cancer were my family members. Before cancer, the presence of family members in my life became smaller due to their social lives. But when I was diagnosed with cancer and was receiving treatment, my family members were always supportive and by my side, and I experienced my family’s love as we stuck together in a difficult situation.

P12 (5.77894) agreed the most with Q38, explaining:

I think mental care is absolutely necessary. After being diagnosed with cancer, I experienced depression, and due to my flawed thinking, I had a hard time understanding myself. I believe that counselling and coaching can expand shrunk thinking and provide mental care. After changing my thinking, I always found that I knew the answer.

Meanwhile, P1 (10.35289), with the highest factor weight for Type 3, and P12 (5.77894), with a significant factor weight, disagreed the most with statement Q15. P1 stated:

I came to know that there really are many people on whom cancer patients can rely and so many social programmes that can support cancer patients. Patients who had the same experience helped and supported each other at events, and the government was operating various programmes for cancer patients through the Cancer Integrated Support Center. I also received a lot of help.

In addition, P12 said, I had quite a few places where I could get help, and I did not feel lonely at all. However, this can vary greatly depending on each individual’s relationship pool and the amount of information they have.

#### 3.2.4. Type 4: A Blessing in Disguise for Post-Traumatic Growth

Type 4 is the “blessing in disguise of post-traumatic growth” type. Type 4 participants perceived their cancer experience as a newly acquired resource, expressing that the time they spent fighting the disease allowed them to grow. They recognised that the most important thing in life was themselves and that an important aspect of social reintegration was to avoid limiting their possibilities. In addition, they recognised that physical health was an important element of social reintegration.

Per Table 5, Type 4 participants showed the strongest agreement with Q13 (“The experience of fighting cancer is another asset”) (z = 1.941), followed by Q30 (“I think the most important thing in social reintegration is physical strength”) (z = 1.491), Q14 (“The cancer became a blessing in disguise because I was able to study and obtain a certificate during the follow-up period”) (z = 1.357), and Q32 (“I feel like I can make choices and live a life better for myself than before I got sick”) (z = 1.235). On the other hand, they showed the strongest disagreement with Q19 (“I wish people did not know the fact that I was sick”) (z = −2.212), followed by Q36 (“I feel really hopeless because there seems to be no hope”) (z = −1.908) and Q17 (“I feel like I am an outcast from society”) (z = −1.644).

P11 (13.20113), representing Type 4 with a high factor weight, agreed the most strongly with statement Q13. She provided the following reason:

Although I lost something due to cancer, I think I have gained a lot. Of course, I get stressed a lot when I feel living restrictions and reach mental limitations, but thanks to the experience, I have accumulated while overcoming the psychological pressure, my threshold has increased, and I think I am obtaining the physical strength needed to endure a little longer in relationships and at work. In this regard, I think my experience with cancer is an invaluable asset that I gained by investing a huge amount of money and physical strength.

P5 (5.90459), who had a meaningful factor weight, agreed the most with Q32, explaining:

Before I got sick, I seemed to have lived a busy life every day with the pleasures of the present rather than for my health or future life. I think I have been living an unhealthy life without feeling the signals my body sends. But after I got cancer, I began to react sensitively to the signals my body sent and acted accordingly, focusing on myself rather than my work or friends while eating well and exercising regularly.

P7 (3.84598), who had a significant factor weight, disagreed the most strongly with Q17. She stated:

During treatment, I felt like I was an outcast, but as I began to come across many cancer patients, cancer survivors, and young cancer patients on YouTube and TV, it seemed that I became a valuable member of a society that exists for people who might get cancer at my age.

P11 (13.20113) and P5 (5.90459) most commonly disagreed with Q19 and explained their reasons as follows:

When I was in college, I agreed with this statement the most. However, in my life, I have come to realise that it does not matter much to people in the private sphere outside of work, so I think that I am now comfortable telling people that I am a cancer patient. However, I also think that I should hide my illness when I apply for a job and think about whether I should work or not, but in the end, I think it is better to be honest and tell the truth in order to combine hospital treatment and work efficiently. (P11)

The first thing my father told me was that I should not hide my illness but should tell people about it so they can learn more about it and get more help. And for me, who was very conscious of ‘how others see me’ and wanted to live a full life without lacking anything, I think disclosing my illness gave me the opportunity to find more time for myself. I was able to say to myself, it is okay to be a little slow and a little lacking. (P5)

Table 6 summarises the key characteristics of the four types.

The perceptions of social reintegration among young adult cancer survivors in their 20s and 30s, based on the conceptual components of reintegration proposed by Ore et al. [43], are shown in Table 7.

## 4. Discussion

This study applied the Q methodology to categorise the perceptions of social reintegration of cancer survivors in their 20s and 30s and examined their characteristics by type. We identified and classified four types based on their main characteristics. Type 1 is “recovery of presence through social reintegration seeking”, Type 2 is “confusion in social reintegration due to social prejudices”, Type 3 is “valuing psychosocial support in the process of social reintegration”, and Type 4 participants see their cancer experience as a “blessing in disguise of post-traumatic growth”.

Type 1 participants expressed that social reintegration means recovery as a main agent of socio-economic activities and serving as a member of society by returning to work. Previous studies indicate that cancer survivors’ return to work enables them to regain normality and control over their lives, thereby increasing their economic stability and self-esteem [55,97]. Type 1 participants had a positive attitude towards social reintegration, which motivated them to take on new challenges. However, they had concerns about the stress they would experience during the employment preparation process for returning to work and the adaptation process after attaining employment; specifically, they feared cancer recurrence. Muzzin et al. [98] stated that cancer survivors experience damaged self-identities and feel a painful sense of loss of normality, integrity, and control over their lives. This explanation helps in understanding Type 1’s characteristics.

Additionally, Type 1 participants wanted to prove themselves through social reintegration. However, they also felt ambivalent about whether they could return to work and take on new challenges. Cancer survivors begin to experience the loss of their health and employment [99]. The disease disrupts their lives, making them change their perspectives of themselves [100]. That is, Type 1 cancer survivors go through a process of re-evaluating and negotiating their identities as patients and survivors, confirming their presence by returning to work, and hoping to recover their self-identities [101,102].

Among Type 1 participants, three had stage 2 cancer, and one had stage 3. Therefore, Type 1 and Type 3 participants were in similar tumour stages. While Type 3 participants felt anxious about social reintegration and wanted psychosocial support and support through relationships, Type 1 participants prioritised challenges to social reintegration and employment. They also wanted social support (e.g., help with job searches, stress management methods, and programmes to create good habits). The content of P3’s post-interview supports this:

I think it would be good if activities to find aptitudes and jobs that would help people overcome career disruptions and find new jobs were created. I think activities that inform people how to manage stress when returning to work would be helpful.

This comment suggests that Type 1 participants need customised support systems to help them recover their self-and occupational identities, support their mental and physical stability, and manage stress. Kim and Tak [103] reported that a career-based competency analysis coaching programme focusing on identifying individuals’ capabilities positively affected the challenges, concreteness, and autonomy of career identity and planning. Above all, institutional support for the social reintegration of cancer survivors should take precedence.

Cancer survivors surveyed by the Korean Cancer Society [104] ranked return-to-work preparation programmes as the most important support measure, with a response ratio of 52.9%. Including cancer survivors in the mandatory employment system for disabled people ranked second, with a response ratio of 50.5%. The European Agency for Safety and Health at Work’s Recurrence and Return to Work after Cancer–Methods and Practices project, Denmark’s local government-based vocational rehabilitation program, and the U.K.’s Macmillan Cancer Support Center’s Overcome Cancer and Work programme are good examples of such initiatives [105,106].

Therefore, the government and companies need to strengthen institutional support, such as employment guides and flexible work arrangements for young cancer survivors, to facilitate their smooth reintegration into society. Additionally, for young individuals like those in Type 1 who are actively seeking to return to work, support programmes such as career coaching, reemployment preparation, and job training are essential. Integrated counselling and coaching programmes could also help manage ambivalent emotions by reducing anxieties and psychological burdens related to social reintegration while supporting the maintenance and management of physical health.

Type 2 participants, classified as “confusion in social reintegration due to social prejudices”, had concerns about receiving unwanted sympathy and facing discrimination during their social reintegration due to society’s negative perceptions of cancer. They had higher sensitivity to social attention than other participants and did not want to reveal that they were fighting cancer. These results align with several previous studies reporting that young cancer survivors fear discrimination, prejudice, and social exclusion and are concerned about becoming the object of excessive sympathy or pity [107,108,109]. The Korean Cancer Society [104] stated that cancer survivors in their 20s and 30s have relatively high levels of discomfort with prejudice and discrimination and tend to respond more sensitively to prejudice and discrimination than survivors in other age groups. The Korean Cancer Society also reported that negative social reintegration cases, such as other cancer survivors’ difficulties in engaging in economic activities or being rejected from jobs, severely affected these cancer survivors psychologically.

These results align with the characteristics of the Type 2 patients in the current study. Type 2 participants expressed scepticism about dating and marriage, indicating that they “want to do it, but it seems difficult”. This viewpoint is similar to previous findings indicating that people with a cancer history are less likely to get married than those without a cancer history [110], and they find it difficult to start new romantic relationships [111].

The demographic characteristics of Type 2 participants indicate that they experienced social reintegration within six months of receiving their treatment, which is faster than other participants. They may have had a lower stage of cancer than other types or a type of cancer from which they could recover more quickly. Moreover, Type 2 participants may have achieved social reintegration quickly because the disease-fighting process was shorter than other types. However, they showed confusion in recognising themselves as different from before and reintegrating into society. They were highly concerned about being exposed to the social perceptions, prejudices, and discrimination associated with cancer; they also demonstrated ambivalence, as they expressed a desire to be socially connected and feel a sense of belonging. Therefore, Type 2 participants require social and cultural support to feel psychologically stable and connect with society safely.

Therefore, Type 2 requires awareness-raising education and campaigns to reduce social prejudice against cancer survivors. Additionally, social relationship support programmes are essential to help them build new connections, feel psychological stability and a sense of belonging, and adapt smoothly to society. Mah et al. [112] indicated that social connectedness is a basic human need and a dimension of belonging. They reported that the social connectedness of young cancer survivors aids post-traumatic growth. In addition, improving cancer survivors’ perceptions requires social effort [113]. Iannarino et al. [114] stated that cancer survivors in their 20s and 30s exhibited an especially strong desire to be seen as “normal” instead of an object of sympathy or discrimination. Positive attention and support encouraged these cancer survivors to perceive and maintain normality, which made them feel relieved.

There is also a need to break away from the misperception that those with diseases will contribute less to the labour force than others and to help the general public understand that anyone can get sick, return to society, and work after recovery despite their illness [115]. To this end, as in countries such as the United Kingdom and Denmark, a collaborative process of various stakeholders such as employers, cancer patients and their guardians, health and social welfare experts, psychological counsellors, and coaches is necessary for the successful social reintegration of cancer survivors in South Korea [105]. In addition, workplaces and organisations must improve their organisational cultures to foster more positive perceptions of cancer survivors [116].

Given that Type 2 individuals feel burdened by disclosing their cancer history, providing anonymous counselling and coaching services is likely to be effective. Additionally, since Type 2 places great importance on family, programmes that enhance communication with family members and promote mutual encouragement and support could help them prepare for social reintegration within a stable psychological environment.

Type 3’s classification is “valuing psychosocial support in the process of social reintegration”. This type can control their fear and anxiety about the possibility of cancer recurrence after they rejoin society. They emphasised the need for mental care in psychological aspects of their lives, such as stress management and emotional control, and aspects of growth, such as creating habits for a healthy life, expanding shrunk thinking, and changing perspectives. Andrykowski et al. [117] stated that psychological health is important for the psychological growth and well-being of cancer survivors. Stan et al. [118] and Berzins et al. [119] reported that wellness coaching improved cancer survivors’ physical and emotional well-being and their quality of life.

Type 3 participants expressed the importance of receiving psychological support through meaningful relationships with family members and friends. Zebrack’s and Isaacson’s [120] study supports this viewpoint. Galán et al. [121] and Okamura et al. [122] also reported that young cancer survivors seek social support from family members and friends. They stated that this characteristic distinguishes young cancer survivors from cancer survivors in other age groups. In addition, Type 3 participants perceived that they needed help from medical professionals with an understanding of cancer and experts who could provide psychological support. Presumably, this need reflects a desire for empathy and to talk about the difficulties of living as a cancer survivor rather than a requirement for professional counselling and treatment. P8’s post-interview supports this notion: “*I think it would help me mentally if I could talk about my difficulties because matters regarding diseases don’t get much sympathy even if we tell them to the general public*”.

Meanwhile, Type 3 participants saw the experience of fighting cancer as an asset because it helped them re-establish relationships and achieve social reintegration. P12’s statement supports this perspective: “*I think it is an asset in that it can reliably filter out people*”. As such, Type 3 participants feel that psychosocial support for growth and recovery is important for social reintegration, with “relationships” being the most vital aspect.

Empowering Type 3 cancer survivors to become the main agents in their wellness journeys is essential. Alongside psychological counselling, they should set and pursue wellness goals. To this end, wellness coaching can provide vital support, helping them shift their perspective, discover their strengths, develop a personalised action plan, and take responsibility for their well-being [118]. Previous international studies support this idea, indicating that wellness coaching positively impacts cancer survivors’ quality of life and overall well-being, including physical, emotional, and functional aspects [118,119,123]. In addition, support is necessary to form social networks, such as self-help groups and cancer survivor mentoring programmes.

A study [117] found that people with supportive interpersonal resources are better able than others to cope with stress by expressing their cancer experiences cognitively and emotionally. Conversely, if social constraints hinder the coping process, a person’s psychological health may worsen. This research has implications for the characteristics of Type 3 cancer survivors. In addition, Park Miree et al. [85] indicated cancer survivors’ need for professional self-help groups and online and offline communities. Another study showed that cancer survivors’ roles as mentors and mentees led to emotional recovery [48,124].

Thus, for Type 3, which emphasises psychosocial support, it is essential to provide wellness coaching programmes focused on stress management, emotional stability, and personal growth. Open communication opportunities, such as support groups and mentoring programmes, should also be made available, allowing individuals to share experiences and receive mutual support.

Type 4 participants see their cancer experience as a “blessing in disguise of post-traumatic growth”. They perceived their cancer experience as a new resource, believing that, although they lost much due to their disease, they also gained much. They also recognised that, although there were psychological pressures and physical difficulties, the experience they accumulated while they overcame these difficulties became a great asset in their social reintegration process. Karlsson et al. [125] indicated that the personal growth experience can create a new attitude towards life, which aligns with the characteristics of Type 4. In addition, Type 4 participants continued their desired activities and learning while fighting the disease and undergoing rehabilitation. They went beyond the stage of accepting changes in their sick bodies and making an effort to restore normalcy. They showed their figure, including changes in their disease experience, and evolved into changed selves. The characteristics of Type 4 participants align with the characteristics of post-traumatic growth that go beyond the previous level of recovery and help survivors undergo positive psychological changes and become healthy and mature [126].

Turner and Cox [127] discovered the themes of “power of will” and “changed perspective” in a study examining participants’ perspectives of their post-traumatic growth experiences. They included “self-understanding” as a sub-theme of the changed perspective, and the participants stated that they changed their perspectives of themselves and the world. They re-established their priorities as they re-evaluated their direction in life and its meaning [128]. P7, who belongs to Type 4, stated:

Before I got sick, I fit myself into my family and the people around me a lot, and I was not very satisfied with my current life. After I got sick, I began to live happily while being satisfied with small things, such as going for a walk or buying flowers for myself.

P5 also described, “I think I began to think and act by thinking of myself as a priority”.

Compared to other participants, Type 4 participants had a higher stage of chronic or stage 4 cancer, which requires long-term treatment and social reintegration. According to Marziliano et al. [25], the cancer stage can determine the relationship between growth and stress. Their studies indicate that the desire for growth strengthens as stress increases, showing similarities to such characteristics.

However, trauma does not lead to positive changes for everyone [30]. Post-traumatic growth does not come from a traumatic event per se but from within oneself, such as from one’s awareness and reflections on oneself after experiencing trauma [129]. In the interviews, Type 4 participants with post-traumatic growth characteristics stated that it would be nice if activities—such as group counselling programmes for social reintegration, lectures to help people control their minds and maintain self-esteem, psychological support and employment preparation classes, and support for settlement after employment—were available. This finding suggests the need for processes and support to enable those who show post-traumatic growth to continue making positive psychological changes.

Therefore, Type 4 participants would benefit from counsellors and coaches who use positive psychology-based approaches to foster post-traumatic growth, which is essential for linking people to positive resources during the social reintegration process. Positive psychology interventions effectively promote well-being, post-traumatic growth, and positive mental health in cancer survivors [126,130].

In addition, Type 4 participants recognised the importance of keeping all their options open. Park et al. [126] emphasised the need to develop programmes to help people identify new possibilities and establish and practise strategic plans because such possibilities are important for achieving post-traumatic growth. This finding shows that positive psychology-based strengths coaching can be an excellent approach.

Thus, for Type 4, which perceives the cancer experience as an opportunity for growth, a coaching approach based on positive psychology that supports self-development and fosters growth is likely to be effective. Additionally, as Type 4 individuals experience positive changes through their cancer journey, motivational activities such as speaking engagements, mentoring programmes, and social media initiatives—allowing them to inspire others—would support a more successful reintegration if provided alongside programmes to help them prepare for these activities.

Suggestions for policy changes based on the results of this study are as follows. First, support approaches according to type are necessary, given that cancer survivors in their 20s and 30s have different characteristics based on their perceptions of social reintegration. Type 1 participants place great importance on restoring social normality and seek to restore their identity by returning to work. Type 3 participants have similar demographic background characteristics as Type 1 participants but recognise that psychosocial support and supportive relationships are important for social reintegration. Type 2 participants have concerns about social reintegration due to social prejudice against cancer patients, while Type 3 participants want to be socially connected. Type 4 participants experience growth through their cancer experiences and view these experiences as another resource for social reintegration. As such, cancer is not a single event but consists of multiple chronic traumas, and persons at all stages of the cancer trajectory can experience different forms of trauma [25]. Therefore, programmes reflecting the subjective perceptions of social reintegration among cancer survivors in their 20s and 30s can be good models for social reintegration programmes for these individuals.

Second, we need a process to enable cancer survivors in their 20s and 30s to overcome the identity crisis caused by cancer. People establish their identities, which is a key developmental task in one’s life, while in their 20s and 30s [131,132]. Notably, the current results show that cancer survivors in their 20s and 30s re-evaluated their identities [97] or experienced identity confusion [133] due to their cancer experiences. We observed these characteristics in the ambivalent perceptions of social reintegration among Type 1 and 3 participants. Type 3 and 4 participants expressed a desire to accept changes in their identity and recover from their traumatic past.

According to Kim et al. [128], it is necessary to prevent the development of post-traumatic stress disorder and guide cancer survivors in their 20s and 30s in adopting a balanced perspective on factors that can help them experience positive changes. This process helps these individuals recall adverse events experienced in the past as positive and meaningful and look forward to future possibilities. In addition, Eichas et al. [134] stated that sharing one’s life story, discovering individual strengths, jointly setting life goals, and establishing strategies to achieve such goals in a group help people form healthy identities. Therefore, support systems could enable cancer survivors in their 20s and 30s to accept changes in their identities due to illness, integrate their disease-related experiences into new identities, and form healthy identities.

Third, an integrated multidisciplinary approach is necessary for the social reintegration of cancer survivors in their 20s and 30s. To achieve social reintegration, they require support for their physical condition, living habits, and psychosocial, cognitive, and spiritual well-being. Therefore, we require integrated information and practical, psychosocial, policy, and institutional support. Moreover, the government should implement a multidisciplinary approach that includes medical care, social welfare, nursing, counselling, and coaching [9]. The main form of psychological support that exists today for cancer survivors is psychotherapy-based counselling. However, positive effects would likely occur if institutions implemented a coaching approach that empowers cancer survivors, awakens their potential, and encourages them to set and achieve goals by themselves, thus enabling them to become the main agents for change in their lives.

Berzins et al. [119] suggested that health coaching is an important tool for cancer survival. In addition, cancer survivors need various approaches for their social reintegration. These programmes should support cancer survivors’ physical well-being, living habits, and spiritual, psychological, and socio-emotional well-being [9]. The connection of the healing industry, which aims to increase people’s psychological, social, cognitive, and physical wellness and can explore connectivity with careers, could bolster existing support methods.

## 5. Conclusions

There has been significant discussion regarding the importance and necessity of cancer survivors’ return to society. However, upon reviewing previous studies, we found that research primarily focused on cancer survivors’ return to work rather than exploring social reintegration from an integrated perspective. Above all, no research has examined the subjective perceptions of young cancer survivors in their 20s and 30s regarding their social reintegration. Therefore, this study investigated the subjective perceptions of young adult cancer survivors in this age group concerning their social reintegration.

Zebrack and Isaacson [120] highlighted that understanding the unique characteristics and shared norms of cancer survivors is essential for discerning their behaviours, attitudes, beliefs, and stress responses. In this study, we conducted an in-depth analysis of the subjective perceptions of young adult cancer survivors and identified four distinct types. According to the findings, young cancer survivors perceive social reintegration not solely as an economic or occupational return but as an integrated concept encompassing physical, psychological, and social dimensions—notably, all four types regarded psychological aspects as a critical factor in their social reintegration. However, we also found distinct differences by type in their motivations for social reintegration, perspectives on reintegration, attitudes towards their cancer experience, health awareness, support needs, and social relationships. Furthermore, based on the definition of the concept of social reintegration by Ore et al. [43], we examined perceptions more multidimensionally by categorising them into psychological, physical, social, economic, and occupational dimensions, as identified in this study.

This study holds academic significance in shedding light on the perspectives and hidden views of young cancer survivors in their 20s and 30s, a group often overlooked concerning their return to society. Practically, this study provides foundational data for developing models and counselling and coaching services tailored to the unique reintegration needs of young cancer survivors according to each type’s characteristics.

However, this study has several limitations. First, as a subjective study involving a limited number of participants, its findings cannot be generalised to all cancer survivors in their 20s and 30s. Second, despite efforts to balance gender and various cancer types to enhance sample representativeness, we did not achieve a gender balance. Therefore, follow-up studies should secure greater gender representation and include quantitative research that addresses factors influencing young cancer survivors’ social reintegration, such as gender, age, and region, more comprehensively.

## Figures and Tables

**Table 1:** Q methodology procedure.

Steps	Research Procedures
Q Population	Literature research
	Internet data search
	In-depth interviews
Q Sample Selection	Primary: Researchers (unstructured sample method)
	Secondary: one cancer survivor in their 20s, one cancer survivor in their 30s
	Tertiary: Three Q methodology experts
	40 Q samples
P Sample Organisation	12 young adult cancer survivors in their 20s and 30s
	Purposive sampling
Q Sample Sorting	Forced distribution by P sample
	11-point scale
Data Analysis	Ken-Q Analysis program
	Factor extraction and rotation: principal component analysis (PCA), Varimax rotation
Interpretation and Typing	Type characterization
	Type naming

**Table 2:** Eigenvalues and explanatory variables of the four types.

Factor	Factor 1	Factor 2	Factor 3	Factor 4
Eigenvalues	3.522128	1.788463	1.528208	1.040372
Explained Variance (%)	29	15	13	9
Cumulative Explained Variance (%)	29	44	57	66

**Table 3:** Correlations between types.

Type	1	2	3	4
1	1	0.044	0.1962	0.3267
2		1	0.2724	0.1791
3			1	0.3686
4				1

**Table 4:** P samples and factor weights of each of the four types.

**Type**	P Sample No.	Loading	Sex	Age (International)	Cancer Type	Cancer Stage	Social Reintegration Period After Cancer Onset	Employment Status	Return to the Same Work
1 (N = 4)	P3	10	F	27	Blood cancer (lymphoma)	Stage 2	Less than 6 months after completion of treatment	N	N
	P10	8.90678	F	35	Breast cancer	Stage 2	More than 1 year after completion of treatment	Y	N
	P2	6.64967	F	29	Breast cancer	Stage 3	More than 1 year after completion of treatment	Y	N
					Thyroid cancer	Before surgery			
	P9	4.49717	F	37	Malignant phyllode tumour	Stage 2	More than 1 year after completion of treatment	Y	N
2 (N = 2)	P6	28.07677	F	28	Thyroid cancer	Unknown	Less than 6 months after completion of treatment	Y	Y
	P4	10.23293	F	33	Breast cancer	Stage 1	Treatment and social reintegration are in parallel	Y	N
3 (N = 3)	P1	10.35289	F	35	Breast cancer	Stage 2	More than 1 year after completion of treatment	Y	N
	P8	6.34935	M	37	Thyroid cancer	Stage 2	Less than 6 months after completion of treatment	Y	Y
	P12	5.77894	F	36	Salivary gland cancer (parotid gland)	Stage 3	More than 1 year after completion of treatment	Y	N
4 (N = 3)	P11	13.20113	F	27	Soft tissue sarcoma cancer	Stage 4	Treatment and social reintegration are in parallel	Y	N
	P5	5.90459	F	35	Blood cancer (chronic myeloid leukaemia)	Chronic stage	Treatment and social reintegration are in parallel	Y	N
	P7	3.84598	F	28	Breast cancer	Stage 1	More than 1 year after completion of treatment	Y	N

**Table 5:** Factor scores with corresponding ranks.

No	Category	Statement	Factor 1		Factor 2		Factor 3		Factor 4	
			Z-Score	Rank	Z-Score	Rank	Z-Score	Rank	Z-Score	Rank
1	Economic professional	I am anxious because I do not properly carry out economic activities compared to my peers.	0.53	15	−0.95	32	−1.94	39	−0.03	24
2		I feel pathetic for having to rely on my parents at an age when I should be independent.	−1.18	34	0.18	18	−1.63	38	−1.48	37
3		I am more stressed because medical expenses are too high when I cannot find a job.	−0.86	29	−1.37	36	−0.24	25	0.55	14
4		I envy my friends who are building their careers while working at companies.	0.97	6	−1.48	38	−0.67	28	0.3	18
5		I am afraid that I may not be promoted or may be pushed out of the front line after returning to work.	−1.23	35	0.48	12	−0.76	30	−1.36	35
6		It is difficult because I cannot secure time for regular checkups while working at a company.	−1.33	36	1.08	6	0.51	10	−0.39	25
7		I feel burdened by the long gap due to cancer treatment.	0.32	16	0.66	9	0.06	22	0.98	8
8		I am worried about whether I should reveal my cancer history or hide it when I resume my work.	0.71	12	1.69	3	−0.5	27	0.51	15
9		I am not sure whether there is a workplace where I can work.	0.11	20	−1.19	34	−0.77	31	−0.96	34
10		I am sorry to my family members because I seem to impose economic and mental burdens on them.	−0.86	30	0.18	19	0.07	20	0.88	9
11	Society (relationships)	I came to know the preciousness of family.	1.01	5	1.98	1	1.55	4	1.19	5
12		I am encouraged by the consideration and support of those around me.	0.28	17	0.42	15	1.53	5	1	7
13		The experience of fighting cancer is another asset.	−0.16	25	−0.26	24	1.48	6	1.94	1
14		The cancer became a blessing in disguise because I was able to study and obtain a certificate during the follow-up period.	−0.88	31	0.32	17	−0.88	34	1.36	3
15		I feel lonely because there is no place to get help.	−0.04	24	−0.32	29	−2.01	40	−0.51	27
16		I want to date and get married, but it seems difficult.	−0.97	33	1.59	4	−0.78	32	0.66	12
17		I feel like I am an outcast from society.	0.84	8	−1.4	37	−0.97	35	−1.64	38
18		I am nervous and anxious because my peers seem to get ahead of me, leaving only me behind.	0.6	14	−1.3	35	−0.83	33	0.11	20
19		I wish people did not know the fact that I was sick.	−0.97	32	1.88	2	−1.29	37	−2.21	40
20		I think the most important thing in social reintegration is information.	0.81	9	0.5	10	0.69	9	−0.53	28
21		I need help because having an open mind myself is difficult.	−0.04	23	0.69	8	0.77	7	−0.83	33
22		Institutional supplementation to enable social reintegration is desperately necessary.	1.71	2	−0.29	25	0.42	13	0.75	10
23	Physical	I am afraid that if I become stressed after social reintegration, the cancer will relapse or metastasise.	2.08	1	1.27	5	1.76	2	−0.03	22
24		I am reluctant to make an appointment because I do not know when and how my condition (physical condition) will change.	0.19	19	−0.58	30	0.27	16	−0.64	30
25		I have difficulties with my sex life, but there is no place where I can get help or talk about it comfortably.	−0.84	28	−1.88	39	−1.16	36	0.42	16
26		I am experiencing psychological difficulties due to infertility and decreased fertility.	−0.53	27	−1.88	40	0.38	14	−1.43	36
27		My confidence in my appearance is dropping.	−1.61	40	0.98	7	0.07	21	−0.03	23
28		I feel that my professional ability has decreased compared to before I became sick.	0.02	22	−0.79	31	0.5	11	−0.49	26
29		I feel shrinkage due to my physical condition being different from before.	0.77	10	0.11	20	0.5	12	0.24	19
30		I think the most important thing in social reintegration is physical strength.	0.68	13	0.4	16	0.7	8	1.49	2
31	Psychology	I feel like I was newly born again.	−1.59	38	−0.29	26	1.69	3	0.66	13
32		I feel like I can make choices and live a better life versus before I got sick.	0.92	7	−0.29	27	0.25	18	1.24	4
33		I think I have a prejudice against cancer.	−1.46	37	0	22	0.09	19	−0.64	31
34		I am at a loss because I have more days to live than the days I have lived.	−0.51	26	−0.29	28	0.35	15	−0.58	29
35		I am solitary and lonely.	0.05	21	−0.21	23	−0.76	29	−0.69	32
36		I feel really hopeless because there seems to be no hope.	−1.59	39	−1.11	33	−0.19	24	−1.91	39
37		I have an ambivalent feeling that I can do it, but I cannot do it (return to work, new challenges, etc.).	1.36	4	0.48	13	0.26	17	0.3	17
38		I need mental care.	0.26	18	0.48	14	1.98	1	0.09	21
39		It is important to think about myself with sufficiently many possibilities opened.	0.77	11	0.5	11	−0.14	23	1.03	6
40		I would like to prove myself thinking that ‘I am still okay’ while working.	1.68	3	0.03	21	−0.37	26	0.69	11

**Table 6:** Comparison of the key characteristics of the four types.

Aspect	Type 1: Recovery of Presence Through Social Reintegration Seeking	Type 2: Confusion in Social Reintegration due to Social Prejudices	Type 3: Valuing Psychosocial Support in the Social Reintegration Process	Type 4: Blessing in Disguise for Post-Traumatic Growth
Primary Motivation	Seeks presence and belonging through social reintegration, particularly work reintegration.	Wants to reintegrate but is highly affected by social prejudices and perceptions.	Prioritises psychological well-being and support throughout reintegration.	Seeks personal growth and values the cancer experience as a transformative asset.
View on Social Reintegration	Views reintegration as a restoration of normalcy and a challenge.	Wants social belonging but feels hindered by stigma and prejudices.	Emphasises the process of reintegration, focusing on mental stability over outcomes.	Views reintegration as an opportunity to enhance personal and physical well-being.
Concerns About Health	Fears recurrence or metastasis due to stress post-reintegration.	Worries about revealing cancer history due to fear of negative perception.	Concerned about stress affecting mental health and cancer relapse.	Believes in maintaining health proactively to support reintegration.
Need for Support	Strongly desires institutional support for reintegration.	Values family support highly but is concerned about societal views.	Strong need for emotional and psychological support from family and friends.	Sees minimal need for societal support, focusing on self-sufficiency.
Attitudes towards Cancer Experience	See cancer experience as a setback; seeks to ‘prove’ normalcy and capability.	Cancer experience creates concern for self-image and acceptance in society.	Sees the cancer experience as a journey requiring mental resilience and support.	Views cancer as a resource and a catalyst for positive life changes.
Ambivalence About Social Relationships	Feels ambivalent due to health concerns but ultimately values social reintegration.	Feels torn between wanting inclusion in society and fearing societal judgment.	Values family connections but may feel independent from societal expectations.	Feels less concerned with societal acceptance; focuses on self-acceptance and growth.

**Table 7:** Individual types and division of dimensions.

	Physical	Psychology	Society (Relationships)	Economic Professional
Type 1: Recovery of Presence Through Social Reintegration Seeking	Normal	Normal	High	High
Type 2: Confusion in Social Reintegration due to Social Prejudices	Low	High	High	Normal
Type 3: Valuing Psychosocial Support in the Social Reintegration Process	Low	High	Normal	Low
Type 4: Blessing in Disguise for Post-Traumatic Growth	High	High	Normal	Normal

## Data Availability

The data are available from the corresponding author upon reasonable request.
